# Coverage and Vaccine Hesitancy of Influenza Vaccination Among Reproductive-Age Women (18–49 Years Old) in China: A National Cross-Sectional Study

**DOI:** 10.3390/vaccines13070752

**Published:** 2025-07-14

**Authors:** Jie Deng, Chenyuan Qin, Min Liu, Jue Liu

**Affiliations:** 1 Department of Epidemiology and Biostatistics, School of Public Health, Peking University, No. 38, Xueyuan Road, Haidian District, Beijing 100191, China; ddajie@pku.edu.cn (J.D.); qincy@bjmu.edu.cn (C.Q.); liumin@bjmu.edu.cn (M.L.); 2 Key Laboratory of Epidemiology of Major Diseases (Peking University), Ministry of Education, No. 38, Xueyuan Road, Haidian District, Beijing 100191, China; 3 Institute for Global Health and Development, Peking University, No. 5 Yiheyuan Road, Haidian District, Beijing 100871, China

**Keywords:** influenza, vaccination, coverage, vaccine hesitancy, reproductive-age women

## Abstract

**Background**: Influenza is a significant global respiratory infection, and vaccinating reproductive-age women, particularly in densely populated countries like China, cannot be overlooked. In this study, we aimed to determine influenza vaccination coverage, vaccine hesitancy, as well as associated factors among Chinese women aged 18–49 years old. **Methods**: A cross-sectional survey among women aged 18–49 years was conducted in China from 15 to 30 March 2023. We collected information such as past-year influenza vaccination, demographic characteristics, health-related factors, COVID-19-related factors, and perceived susceptibility and severity of influenza. Influenza vaccine acceptance among participants who did not receive influenza vaccination in the past year was also investigated. Multivariable logistic regression analyses were employed to investigate the influencing factors of vaccine coverage and vaccine hesitancy. **Results**: A total of 1742 reproductive-aged women were included in the final analysis. The past-year influenza vaccine coverage among women aged 18–49 years old was only 39.32% in China. Age ≥ 35 years (aOR = 0.72, 95% CI: 0.56–0.94), renting accommodation (aOR = 0.57, 95% CI: 0.44–0.75), and history of COVID-19 infection (aOR = 0.65, 95% CI: 0.47–0.89) and COVID-19 vaccine hesitancy (aOR = 0.39, 95% CI: 0.29–0.54) were all identified as negative correlates of influenza vaccine coverage among Chinese reproductive-aged women, while participants with a history of chronic diseases (aOR = 1.57, 95% CI: 1.23–2.01) and noticeable pandemic fatigue due to COVID-19 (aOR = 1.45, 95% CI: 1.05–2.00) were prone to have higher vaccination rates. Among reproductive-aged women who did not receive influenza vaccination in the past year, the hesitancy rate regarding future influenza vaccination was 31.79%. Factors such as older age, urban residence, living with others, poor self-rated health status, absence of chronic diseases, completion of full COVID-19 vaccination, COVID-19 vaccine hesitancy, pandemic fatigue, and failure to perceive the susceptibility and severity of influenza might increase influenza vaccine hesitancy. **Discussion**: Overall, a lower coverage rate of influenza vaccine was notably observed among Chinese reproductive-age women, as well as the hesitancy regarding future vaccination. To effectively mitigate the impact of influenza and reduce the incidence of associated diseases, it is imperative to devise targeted intervention strategies and policies tailored to reproductive-age women.

## 1. Introduction

Influenza is one of the most common global respiratory infections, contributing to significant morbidity and mortality each year, particularly among vulnerable populations such as the elderly, young children, pregnant women, and individuals with underlying health conditions [1]. According to the World Health Organization (WHO), there are around a billion cases of seasonal influenza annually, including 3–5 million cases of severe illness, causing 290,000 to 650,000 respiratory deaths, which poses a substantial burden on healthcare systems and public health [1,2]. In China, influenza posed a great public health and economic challenge, with an incidence rate of 5.51 cases per 100,000 population in 2018 and an estimated financial burden of CNY 26.38 billion (USD 3.819 billion) in 2019—86.4% of which stemmed from hospitalization costs [3]. Vaccination is widely recognized as the most effective way to prevent infection and reduce the severity of disease, hospitalizations, and deaths caused by influenza viruses [4].

In China, influenza typically occurred in winter and spring during each monitoring year, peaking from November to March of the next year, with a stable seasonal characteristic of influenza between the 2010/2011 and 2018/2019 seasons before the coronavirus disease 2019 (COVID-19) outbreaks [5]. Cao et al. found that influenza activity decreased in January 2020 (after COVID-19 outbreaks) since intense nonpharmaceutical interventions (NPIs) were implemented to limit the COVID-19 pandemic in China and was historically low through the 2020/2021 season (influenza-positive percentage of 0.7% in southern China and 0.2% in northern China) [5]. Influenza virus circulation resumed from a low epidemic in the 2020/2021 season in southern and northern China and showed an out-of-season epidemic in the first surveillance weeks in the 2022/2023 season in southern China [5]. This resurgence may have influenced public perceptions of flu risk and potentially contributed to lower uptake if vaccination campaigns were seen as mistimed relative to the epidemic curve.

Reproductive-age women aged 18–49 years old represent a critical population for influenza vaccination, not only due to their direct susceptibility but also because of their potential roles as mothers and caregivers who can influence family-level vaccination decisions and protect infants who are not yet eligible for vaccination, underscoring the broader public health impact of targeting this demographic [6,7,8]. Furthermore, influenza infection during pregnancy has been associated with an increased risk of severe adverse pregnancy outcomes, as well as adverse birth outcomes [9]. Given China’s large population and the increasing urbanization and mobility of young women, understanding their vaccination behavior is vital for informing national immunization strategies.

Despite the recognized benefits of influenza vaccination, global and national influenza vaccine uptake rates among adults remain suboptimal. In America, only 47.2% of women received influenza vaccination before or during pregnancy during the 2022–2023 influenza season—a coverage rate > 10 percentage points lower than during the 2019–2020 season, and they were all below the Healthy People 2020 goal of 80.0% [10,11,12]. A study conducted in Singapore revealed low influenza vaccination coverage among pregnant women (9.8% in 2017), with nearly half of unvaccinated women expressing unwillingness to receive the vaccine [6,13]. In Japan, the mean coverage for the seasonal influenza vaccine during pregnancy was 27.0% to 53.5% [12]. In China, despite government efforts to promote influenza vaccination, coverage of influenza vaccination among the Chinese population remains low. A cross-sectional study conducted in Shanghai, China, revealed that the overall influenza vaccination coverage during the 2021–2022 season was only 17.68%, with the highest coverage observed among children (35.68%), followed by adults (12.75%) and elderly populations (11.70%), indicating that Shanghai’s overall influenza vaccination rate remains suboptimal for establishing effective herd immunity and lags behind vaccination levels in other developed regions [14].

Vaccine hesitancy, defined as the reluctance or refusal to vaccinate despite the availability of vaccines, has been recognized by the WHO as one of the ten threats to global health in 2019 [15]. Previous studies have identified multiple barriers to influenza vaccine uptake, such as access issues, lack of awareness, misperceptions about influenza risk, concerns about vaccine safety, and lack of healthcare provider recommendations [12,16,17]. Additionally, the COVID-19 pandemic has introduced new dynamics, such as pandemic fatigue and altered perceptions of vaccination, which may further influence influenza vaccine acceptance [18,19]. Previous studies have highlighted that COVID-19 experiences may either increase general vaccine acceptance or, conversely, fuel hesitancy due to pandemic fatigue and distrust [20]. In China, although seasonal influenza vaccines are available and recommended, they are not included in the National Immunization Program (NIP), meaning individuals often must pay out of pocket, which may further limit the uptake of influenza vaccines [21].

Currently, seasonal influenza infection is still a great public health threat globally, understanding the determinants of both vaccine coverage and hesitancy is essential for designing targeted interventions to improve immunization rates in this population. However, data specifically focusing on influenza vaccine coverage and hesitancy among reproductive-aged women in China are lacking. Therefore, we conducted a cross-sectional study in China from 15–30 March 2023, aiming to determine the coverage and vaccine hesitancy of influenza vaccination, as well as their influencing factors among reproductive-age women. These findings will help provide critical insights to inform targeted public health interventions and policy strategies to improve influenza vaccination uptake among this important population group in China.

## 2. Methods

### 2.1. Study Design and Participants

To evaluate the coverage and vaccine hesitancy of influenza vaccination among reproductive-age women, we conducted an anonymous cross-sectional survey in China from 15–30 March 2023. This online survey was performed by a professional scientific data platform (Changsha Ranxing Information Technology Co., Ltd., Changsha, China) with nearly 300 million users every month [22]. It can accurately send the electronic questionnaire to our expected representative respondents based on the clear personal information (such as age, gender, and residence) of registered members [22]. Furthermore, participants were required to complete all the items before they were able to submit the questionnaire. Recruitment criteria were as follows: (1) agree to fill in the questionnaire carefully; (2) 18–49 years old; and (3) females. Informed consent was embedded, and all respondents provided consent for anonymized data use for academic purposes. Moreover, to ensure data quality, a minimum completion time of 300 s was set for the online questionnaire. Responses submitted faster were excluded to reduce inattentive or random answers and to improve the reliability of the data collected.

According to previous studies, the rates of influenza vaccination and vaccine hesitancy among women in China were 27.7% and 14.5%, respectively [6]. Considering that the coverage rate of influenza vaccine was 27.7% (*p* = 0.0277) [6], with the α set as 0.05 and the confidence interval (CI) width as 0.05*p* (*p* = vaccination rate in this study), the minimum sample size was 1269 when using the exact (Clopper–Pearson) method for calculation. Likewise, the minimum sample size was 801 while considering the rate of influenza vaccine hesitancy as 14.5% [6]. In order to enhance the geographic representativeness of the sample, the quota sampling method was applied to allocate the sample proportionally across 31 provinces based on the population distribution reported in the Seventh National Census. After quality control and manual inspection procedures, 1742 eligible reproductive-age women were enrolled in the final analysis. The PASS software 21.0.3 (NCSS LLC, Kaysville, UT, USA) was used to calculate the sample size.

### 2.2. Measures

#### 2.2.1. Coverage of Influenza Vaccines

Influenza vaccination coverage among reproductive-age women in China was assessed through a self-reported survey item, which asked participants: “Have you been vaccinated against influenza within the past year?” This question was designed to capture whether individuals had received the influenza vaccine within the 12 months prior to the survey, providing a measure of recent vaccine uptake. Respondents were asked to select one of two options: “Yes” or “No”. The coverage of influenza vaccines was calculated as the proportion of individuals who reported receiving the influenza vaccine out of the total number of respondents.

#### 2.2.2. Attitude Toward the Influenza Vaccines

To determine the attitude of participants who did not receive influenza vaccination in the past year towards receiving the influenza vaccines in the future, we set a question as “Are you willing to receive the influenza vaccine in the future?” The answer was progressively set on a 5-point Likert scale (1 = very reluctant, 2 = reluctant, 3 = not sure, 4 = willing, 5 = very willing). We defined vaccine hesitancy as reluctance or uncertainty about receiving the influenza vaccines, and then further asked for specific reasons for hesitancy. Likewise, the attitude of participants towards receiving the next dose of COVID-19 vaccines was also evaluated.

#### 2.2.3. Covariates

Sociodemographic variables and health-related factors were collected in this study as covariables. Sociodemographic variables included age, location, education, marital status, housing status, income level, family members, and having children or not. Health-related factors included subjective health status, chronic disease history, anxiety, depression, COVID-19 infection history, and COVID-19 vaccination history. Chronic disease history indicated a previous diagnosis of one or more of the following diseases: hypertension, dyslipidemia, stroke, heart disease, osteoarthritis/rheumatoid arthritis/osteoporosis, tuberculosis, asthma, diabetes mellitus, cancer, and others. The Generalized Anxiety Disorder 7-item (GAD-7) scale and the Patient Health Questionnaire-9 (PHQ-9) scale were used to assess the respondents’ self-perceived anxiety and depression symptoms, respectively [23,24]. According to previous studies, we defined GAD-7 ≥ 10 as positive for anxiety symptoms and PHQ-9 ≥ 10 as positive for depressive symptoms in this study [25,26]. In addition, we also assessed subjects’ fatigue from the COVID-19 pandemic using the Pandemic Fatigue Scale (PFS) [27]. The PFS is a subjective questionnaire, with a total score ranging from 6 to 42 points. Higher scores indicate more pronounced pandemic fatigue. The levels of pandemic fatigue were divided into “Low (6–18 points)”, “Moderate (19–30 points)”, and “High (31–42 points)”. Risk perception items of influenza were adapted from the health belief model and involved two parts: perceived susceptibility and perceived severity. Participants were asked “How likely do you think you are to be infected with influenza?” and “If you are infected with influenza, how severe do you think it will be?” The 5-point scale answers (1 = very low, 2 = low, 3 = moderate, 4 = high, 5 = very high) were divided into “Low”, “Moderate”, and “High” levels [28].

### 2.3. Statistics Analysis

Descriptive statistics were collected to describe the characteristics and the influenza vaccine coverage rates and hesitancy rates among reproductive-age women, and 95% CIs were also calculated. Continuous variables were described by the mean (M) and standard deviation (SD) and compared by *t*-test or analysis of variance (ANOVA). Categorical variables were described by frequency and percentage and compared across groups by the chi-square test or Fisher’s exact test. Variance inflation factors (VIFs) were used to test for multicollinearity among the related variables, and all VIF values were within the acceptable range. Therefore, multivariable logistic regression analyses were conducted to assess the adjusted associations of factors influencing the coverage and hesitancy of influenza vaccination and were adjusted by age, location, education, marital status, housing, income level, family member, having children or not, subjective health status, chronic disease, anxiety, depression, history of COVID-19 infection and vaccination, hesitancy towards the COVID-19 vaccine, COVID-19 pandemic fatigue, as well as perceived susceptibility and severity of influenza. Adjusted odds ratios (aORs) with 95% CIs for each variable were calculated.

All statistical analyses were conducted by Software SPSS 29.0 (IBM SPSS Inc., New York, NY, USA), and we set the significance level at a two-sided *p*-value of <0.05.

## 3. Results

### 3.1. Characteristics of Participants

A total of 1742 reproductive-age women were included in the final analysis (Table 1). Among all participants, 1419 (81.46%) were <35 years old, 1061 (60.91%) lived in urban areas, 1322 (75.89%) had at least a bachelor’s degree, 1272 (73.02%) had already married, 1415 (81.23%) lived in owner-occupied house, 1624 (93.23%) lived with others (family members ≥ 2), and 1160 (66.59%) had at least one child. In terms of health status, only 69 (3.96%) reported bad subjective health status, 408 (23.42%) struggled with chronic diseases, 448 (25.72%), and 547 (31.40%) were assessed as having anxiety or depression, respectively. In addition, the majority of participants had been infected with COVID-19 (91.50%) and had completed either full COVID-19 vaccination (46.61%) or at least one booster dose (40.59%). As for the attitude toward the COVID-19 vaccine, 1472 (84.50%) were willing to receive the next dose of COVID-19 vaccine. Furthermore, 759 (43.57%) and 211 (12.11%) participants showed moderate or high COVID-19 pandemic fatigue, respectively. Finally, 20.32% of all respondents believed that they had a low risk of influenza, and nearly 70% of them believed that the consequences of influenza would not be severe.

### 3.2. Coverage of Influenza Vaccines

The past-year influenza vaccine coverage among reproductive-age women (18–49 years old) was only 39.32% in China (Table 1). During the past year, married reproductive-age women demonstrated higher influenza vaccine coverage compared to unmarried/divorced/widowed respondents (41.51% vs. 33.40%, *p* = 0.002). Reproductive-age women living in owner-occupied housing were more likely to be vaccinated with influenza vaccines compared to those in rental housing (41.63% vs. 29.36%, *p* < 0.001). Similarly, those with higher income levels had higher influenza vaccine coverage (39.99% vs. 30.65%, *p* = 0.040). Women with children also showed increased influenza vaccine coverage (42.16% vs. 33.68%, *p* < 0.001). Respondents who perceived themselves to have a better health status had higher influenza vaccination rates, with those reporting bad, moderate, and great objective health status showing influenza vaccine coverage of 23.19%, 36.80%, and 41.71%, respectively (*p* = 0.003). It is also observed that women with chronic diseases had a higher influenza vaccine coverage compared to those without any chronic disease (47.06% vs. 36.96%, *p* < 0.001). Respondents who were fully vaccinated with COVID-19 vaccines (42.12%) or vaccinated with at least one booster dose (39.18%) showed higher influenza vaccine coverage (*p* = 0.003). In addition, participants willing to receive COVID-19 vaccines demonstrated higher influenza vaccine coverage (42.32% vs. 22.96%, *p* < 0.001). Participants who perceived the consequences of influenza as more severe had a higher influenza vaccine coverage (46.77%, *p* = 0.003).

### 3.3. Influencing Factors of Influenza Vaccine Coverage

Multivariable logistic regression analyses showed that age ≥ 35 years (aOR = 0.72, 95% CI: 0.56–0.94, *p* = 0.015), renting accommodation (aOR = 0.57, 95% CI: 0.44–0.75, *p* < 001), and history of COVID-19 infection (aOR = 0.65, 95% CI: 0.47–0.89, *p* = 0.006) and COVID-19 vaccine hesitancy (aOR = 0.39, 95% CI: 0.29–0.54, *p* < 0.001) were all identified as negative correlates of influenza vaccine coverage among Chinese reproductive-aged women. While participants with a history of chronic diseases (aOR = 1.57, 95% CI: 1.23–2.01, *p* < 001) and noticeable pandemic fatigue due to COVID-19 (aOR = 1.45, 95% CI: 1.05–2.00, *p* = 0.025) were prone to have higher vaccination rates (Table 2).

### 3.4. Attitude Toward Influenza Vaccines

Among the 1057 reproductive-aged women who did not receive influenza vaccination in the past year, the hesitancy rate regarding future influenza vaccination was 31.79% (*n* = 336). As shown in Table 3, women aged ≥ 35 years exhibited higher hesitancy toward influenza vaccines compared to women aged < 35 years (42.79% vs. 29.09%, *p* < 0.001), and urban residents were more hesitant than rural residents (34.88% vs. 27.31%, *p* = 0.009). Among respondents with different family sizes, those living alone had the highest influenza vaccine hesitancy rate at 39.02% (*p* = 0.003). Notably, respondents who were hesitant about the COVID-19 vaccines exhibited significantly higher hesitancy toward the influenza vaccines, with a hesitancy rate nearly three times higher than those without COVID-19 vaccine hesitancy (66.83% vs. 23.20%, *p* < 0.001). In addition, those who reported high levels of pandemic fatigue toward COVID-19 were more hesitant to receive the influenza vaccine, with a hesitancy rate of 46.49% (*p* < 0.001). Furthermore, hesitancy toward the influenza vaccine was more common among those perceiving low susceptibility of influenza (41.89%, *p* < 0.001) and low severity of influenza (41.25%, *p* < 0.001); more details are shown in Table 3.

### 3.5. Influencing Factors of Influenza Vaccine Hesitancy

Multivariable logistic regression analyses of factors influencing influenza vaccine hesitancy are shown in Table 4. Among reproductive-aged women who did not receive influenza vaccination in the past year, factors such as age ≥ 35 years (aOR = 1.70, 95% CI: 1.18–2.45, *p* = 0.004), living with others (2–3 family members: aOR = 1.78, 95% CI: 1.03–3.10, *p* = 0.040; 4 family members: aOR = 1.44, 95% CI: 1.05–1.97, *p* = 0.023), completion of full COVID-19 vaccination (aOR = 1.59, 95% CI: 1.04–2.42, *p* = 0.033), COVID-19 vaccine hesitancy (aOR = 5.94, 95% CI: 4.11–8.58, *p* < 0.001), and pandemic fatigue (moderate: aOR = 2.14, 95% CI: 1.55–2.96, *p* < 0.001; high: aOR = 2.23, 95% CI: 1.36–3.66, *p* = 0.002) might increase influenza vaccine hesitancy. On the contrary, participants with rural residence (aOR = 0.71, 95% CI: 0.52–0.97, *p* = 0.031), normal or great subjective health status (normal: aOR = 0.36, 95% CI: 0.22–0.61, *p* < 0.001; great: aOR = 0.25, 95% CI: 0.16–0.40, *p* < 0.001), chronic disease (aOR = 0.61, 95% CI: 0.41–0.91, *p* = 0.014), perceived susceptibility to influenza (moderate: aOR = 0.57, 95% CI: 0.39–0.83, *p* = 0.004; high: aOR = 0.46, 95% CI: 0.30–0.72, *p* < 0.001), and perceived severity of influenza (moderate: aOR = 0.67, 95% CI: 0.48–0.95, *p* = 0.023; high: aOR = 0.44, 95% CI: 0.27–0.73, *p* = 0.001) were more willing to receive the influenza vaccine.

## 4. Discussion

In this national cross-sectional study, we found that the past-year influenza vaccine coverage among reproductive-age women (18–49 years old) in China was only 39.32%, and nearly one-third (31.79%) of unvaccinated women expressed hesitancy toward future influenza vaccination. These findings indicate suboptimal influenza vaccination uptake and a substantial level of hesitancy in this important demographic group, which may compromise both maternal and child health, especially during seasonal influenza epidemics. Additionally, we further explored the factors influencing influenza vaccine coverage and acceptance among reproductive-age women, which will help devise targeted intervention strategies and policies tailored to reproductive-age women, to effectively mitigate the impact of influenza and reduce the incidence of associated diseases.

Our study identified several sociodemographic and psychosocial factors associated with influenza vaccine coverage and hesitancy among reproductive-age women in China. Notably, older age (≥35 years), renting accommodation, prior COVID-19 infection, and COVID-19 vaccine hesitancy were associated with lower influenza vaccine coverage, whereas having chronic diseases and noticeable pandemic fatigue due to COVID-19 were linked with higher vaccination uptake. These findings suggest that older reproductive-age women and those with unstable housing conditions may be less engaged with routine preventive healthcare services, potentially due to time constraints, financial concerns, or lower perceived benefits of vaccination. The inverse relationship between COVID-19 infection history and influenza vaccination may reflect a sense of immunity complacency or mistrust in vaccination following COVID-related experiences.

Vaccine hesitancy toward future influenza vaccination among reproductive-age women in China was found to be multifactorial. Reproductive-age women who were hesitant toward COVID-19 vaccination were more likely to express influenza vaccine hesitancy. Lin et al.’s review found that influenza vaccination was an important facilitator for COVID-19 vaccine confidence and acceptance during the COVID-19 pandemic, highlighting a possible spillover effect of vaccine attitudes across different types of vaccines [20,29]. Prior studies also have shown that general vaccine mistrust, fear of side effects, and inconsistent messaging can simultaneously affect attitudes toward multiple vaccines [30]. Pandemic fatigue about COVID-19, although associated with higher influenza vaccine coverage, paradoxically contributed to greater vaccine hesitancy, possibly due to psychological exhaustion or reduced trust in public health messaging over time. Previous studies also showed that pandemic fatigue was positively associated with COVID-19 vaccine hesitation rates among different populations (such as general adults or people who recovered from COVID-19), as well as with parents’ attitudes toward their children [31,32,33]. Furthermore, we found that reproductive-age women with lower perceived susceptibility and severity of influenza were more likely to be hesitant about influenza vaccination, which is consistent with the findings of Du et al. [34]. In their study among reproductive women in China during the COVID-19 pandemic, low perceived susceptibility was significantly associated with increased influenza vaccine hesitancy for their children (aOR = 2.55, 95% CI: 1.79–3.65), along with low perceived benefit and high perceived barriers [34]. These results reinforce the importance of improving risk perception and communicating the tangible benefits of influenza vaccination to enhance acceptance [35,36].

Previous studies have often reported lower vaccine acceptance among younger populations, which contrasted with our findings [37,38,39]. Our study revealed that compared to reproductive-aged women under 35 years old, those aged ≥ 35 years had lower influenza vaccination coverage and higher influenza vaccine hesitancy. One possible explanation is that younger reproductive-aged women have greater exposure to health information through social media, schools, or workplaces, so they might be better informed about disease severity, vaccine safety, and efficacy, facilitating vaccine acceptance [6]. In contrast, older individuals typically engage less with social media for health information [6]. Given that higher age is a major risk factor for severe influenza-related illness and death—and that older pregnant women or infected pregnant women face an increased risk of adverse pregnancy outcomes—this demographic should be prioritized for vaccination [1,40]. Moreover, the role of digital access and online engagement in shaping vaccine attitudes warrants attention. Cosma et al. highlighted that digital tools such as chatbots can improve vaccine literacy and decision-making through credible, interactive communication [41]. Within the increasingly digital health landscape, particularly in China, incorporating such tools into future strategies could enhance influenza vaccine uptake. Therefore, future interventions should consider integrating digital platforms into national and local vaccine promotion campaigns, targeting not only younger cohorts who are digitally literate and receptive to online health messaging, but also older populations, through age-appropriate and accessible digital tools to bridge the digital divide.

Despite the relatively high expressed willingness for influenza vaccination among unvaccinated women, our findings showed a substantial gap between intention and actual vaccine uptake. This disconnect is not unique to China. Del Riccio et al. analyzed influenza vaccination trends in 12 countries and found that although many countries experienced a temporary increase in influenza vaccine uptake during the COVID-19 pandemic, these gains were often not sustained [42]. The short-lived improvement in vaccine uptake during public health crises failed to translate into sustained coverage due to a combination of waning political attention, inconsistent messaging, and a lack of integration of temporary behavioral changes into long-term public health strategies [42]. Similarly, our findings suggested that although reproductive-age women in China showed a considerable intention to receive influenza vaccination, particularly in the wake of heightened pandemic awareness, these intentions might not readily translate into actual uptake in the absence of consistent reinforcement mechanisms. Furthermore, one important structural barrier to influenza vaccine uptake among reproductive-age women in China is probably that the vaccine is not included in the National Immunization Program (NIP). This financial burden may discourage economically dependent or younger women, especially those without prior health-seeking behavior. Additionally, the lack of official endorsement through the NIP may reduce public awareness and perceived necessity of vaccination, as shown in previous studies [43]. These factors highlight a critical need for sustained and structured public health efforts—such as continuous risk communication, regular community outreach, and integration of influenza vaccination into routine health services—to convert short-term awareness into lasting vaccination behavior, particularly among adults who are not systematically included in national immunization programs.

This is the first nationwide study to evaluate the coverage and vaccine hesitancy of influenza vaccination among reproductive-age women during the post-pandemic era of COVID-19 and that encompasses all provinces in mainland China and is representative and statistically efficacious. However, there also some limitations. First, as a cross-sectional study, we cannot claim causality and should also be cautious while interpreting observed associations. Second, the online survey might introduce response bias, as individuals with higher education and better internet access were more likely to participate. The high proportion of respondents with a bachelor’s degree or above may not fully reflect the broader population. In addition, the study relied on self-reported information regarding vaccination status, attitudes, and health conditions, which may be subject to recall bias and social desirability bias. Moreover, we did not assess participants’ current pregnancy status, which could influence vaccination decisions. Although we included whether respondents had children, this might not fully capture the impact of pregnancy on vaccine uptake and attitudes. Future studies should consider including pregnancy status to better understand its potential role in influenza vaccination decisions among women of reproductive age. While our study assessed chronic disease status in general, we did not further distinguish between specific conditions. This decision aimed to minimize survey burden. Future research could explore these differences to inform targeted strategies. Finally, some important factors, such as healthcare access, provider recommendation, and awareness of free or subsidized vaccination policies, were not directly assessed, which could have provided further insight into the determinants of vaccine uptake and hesitancy. Also, given the scope and focus of our study, we prioritized identifying main effects to ensure model parsimony and interpretability. Future research could benefit from examining interaction effects to further elucidate the complex factors influencing influenza vaccination behavior.

## 5. Conclusions

In this study, we found that the past-year influenza vaccine coverage among reproductive-age women in China was only 39.32%, and nearly one-third of unvaccinated women expressed hesitancy toward future influenza vaccination, indicating suboptimal influenza vaccination uptake and a substantial level of hesitancy in this important demographic group, which may compromise both maternal and child health, especially during seasonal influenza epidemics. Additionally, we further explored the factors influencing influenza vaccine coverage and acceptance among reproductive-age women. Participants with chronic diseases and noticeable pandemic fatigue due to COVID-19 were prone to have higher vaccination rates. Furthermore, it is also observed that factors such as older age, urban residence, living with others, poor self-rated health status, absence of chronic diseases, completion of full COVID-19 vaccination, COVID-19 vaccine hesitancy, pandemic fatigue, and failure to perceive the susceptibility and severity of influenza might increase influenza vaccine hesitancy. These findings will help devise targeted intervention strategies and policies tailored to reproductive-age women to effectively mitigate the impact of influenza and reduce the incidence of associated diseases.

## Figures and Tables

**Table 1:** Characteristics and coverage of influenza vaccination among 1742 reproductive-age women in China.

Characteristics	Number (%)	Coverage of Influenza Vaccination		
		*n* (%)	95% CI	*p* Value
Total	1742 (100.00)	685 (39.32)	37.05–41.63	
Age (years)				0.130
<35	1419 (81.46)	570 (40.17)	37.64–42.74	
≥35	323 (18.54)	115 (35.60)	30.53–40.94	
Location				0.059
Urban	1061 (60.91)	436 (41.09)	38.16–44.07	
Rural	681 (39.09)	249 (36.56)	33.01–40.23	
Education				0.216
High school and below	296 (16.99)	103 (34.80)	29.54–40.35	
Bachelor’s degree	1322 (75.89)	532 (40.24)	37.62–42.90	
Master’s degree	124 (7.12)	50 (40.32)	31.99–49.10	
Marital status				0.002 *
Unmarried, divorced, or widowed	470 (26.98)	157 (33.40)	29.25–37.76	
Married	1272 (73.02)	528 (41.51)	38.82–44.23	
Housing status				<0.001 *
Owner-occupied	1415 (81.23)	589 (41.63)	39.08–44.21	
Rental	327 (18.77)	96 (29.36)	24.62–34.46	
Income level				0.040 *
Lower	124 (7.12)	38 (30.65)	23.05–39.13	
Higher	1618 (92.88)	647 (39.99)	37.62–42.39	
Family member				0.065
1	118 (6.77)	36 (30.51)	22.75–39.21	
2–3	919 (52.76)	356 (38.74)	35.63–41.92	
4	705 (40.47)	293 (41.56)	37.96–45.23	
Have children				<0.001 *
No	582 (33.41)	196 (33.68)	29.93–37.59	
Yes	1160 (66.59)	489 (42.16)	39.34–45.01	
Subjective health status				0.003 *
Bad	69 (3.96)	16 (23.19)	14.46–34.11	
Normal	587 (33.70)	216 (36.80)	32.97–40.76	
Great	1086 (62.34)	453 (41.71)	38.81–44.66	
Chronic disease				<0.001 *
No	1334 (76.58)	493 (36.96)	34.40–39.57	
Yes	408 (23.42)	192 (47.06)	42.25–51.91	
Anxiety				0.379
No	1294 (74.28)	501 (38.72)	36.09–41.39	
Yes	448 (25.72)	184 (41.07)	36.59–45.67	
Depression				0.347
No	1195 (68.60)	461 (38.58)	35.85–41.36	
Yes	547 (31.40)	224 (40.95)	36.89–45.11	
History of COVID-19 infection				0.398
No	148 (8.50)	63 (42.57)	34.81–50.61	
Yes	1594 (91.50)	622 (39.02)	36.65–41.43	
History of COVID-19 vaccination				0.003 *
Not fully vaccinated	223 (12.80)	66 (29.60)	23.90–35.82	
Fully vaccinated	812 (46.61)	342 (42.12)	38.76–45.54	
At least one booster dose	707 (40.59)	277 (39.18)	35.63–42.82	
Hesitancy of COVID-19 vaccine				<0.001 *
No	1472 (84.50)	623 (42.32)	39.82–44.86	
Yes	270 (15.50)	62 (22.96)	18.25–28.25	
Pandemic fatigue of COVID-19				0.011 *
Low	772 (44.32)	317 (41.06)	37.63–44.56	
Moderate	759 (43.57)	271 (35.70)	32.36–39.16	
High	211 (12.11)	97 (45.97)	39.34–52.71	
Perceived susceptibility of influenza				0.629
Low	354 (20.32)	132 (37.29)	32.37–42.41	
Moderate	837 (48.05)	337 (40.26)	36.98–43.61	
High	551 (31.63)	216 (39.20)	35.19–43.33	
Perceived severity of influenza				0.003 *
Low	524 (30.08)	204 (38.93)	34.83–43.16	
Moderate	846 (48.56)	307 (36.29)	33.10–39.57	
High	372 (21.35)	174 (46.77)	41.75–51.85	

* A *p*-value less than 0.05 is considered to be statistically significant.

**Table 2:** Influencing factors of influenza vaccine coverage among reproductive-age women in China.

Variables	aOR	95% CI	*p* Value
Age (Ref.: <35 years)			
≥35 years	0.72	0.56–0.94	0.015 *
Housing status (ref.: owner-occupied)			
Rental	0.57	0.44–0.75	<0.001 *
Subjective health status (ref.: bad)			
Normal	1.11	0.78–1.57	0.579
Great	1.36	0.99–1.85	0.055
Chronic disease (ref.: no)			
Yes	1.57	1.23–2.01	<0.001 *
History of COVID-19 infection (ref.: no)			
Yes	0.65	0.47–0.89	0.006 *
Hesitancy of COVID-19 vaccine (ref.: no)			
Yes	0.39	0.29–0.54	<0.001 *
Pandemic fatigue from COVID-19 (ref.: low)			
Moderate	0.88	0.71–1.09	0.243
High	1.45	1.05–2.00	0.025 *
Perceived severity of influenza (ref.: low)			
Moderate	0.84	0.67–1.05	0.128
High	1.16	0.88–1.53	0.286

* A *p*-value less than 0.05 is considered to be statistically significant.

**Table 3:** Characteristics and influenza vaccine hesitancy among 1057 reproductive-age women without past-year influenza vaccination in China.

Characteristics	Number (%)	Hesitancy of Influenza Vaccine		
		*n* (%)	95% CI	*p* Value
Total	1057 (100.00)	336 (31.79)	29.03–34.64	
Age (years)				<0.001 *
<35	849 (80.32)	247 (29.09)	26.11–32.22	
≥35	208 (19.68)	89 (42.79)	36.20–49.57	
Location				0.009 *
Urban	625 (59.13)	218 (34.88)	31.22–38.68	
Rural	432 (40.87)	118 (27.31)	23.27–31.66	
Education				0.133
High school and below	193 (18.26)	63 (32.64)	26.32–39.48	
Bachelor’s degree	790 (74.74)	242 (30.63)	27.49–33.91	
Master’s degree	74 (7.00)	31 (41.89)	31.14–53.27	
Marital status				0.347
Unmarried, divorced, or widowed	313 (29.61)	106 (33.87)	28.79–39.24	
Married	744 (70.39)	230 (30.91)	27.67–34.30	
Housing status				0.294
Owner-occupied	826 (78.15)	256 (30.99)	27.91–34.21	
Rental	231 (21.85)	80 (34.63)	28.72–40.93	
Income level				0.688
Lower	86 (8.14)	29 (33.72)	24.4–44.12	
Higher	971 (91.86)	307 (31.62)	28.75–34.59	
Family member				0.003 *
1	82 (7.76)	32 (39.02)	29.00–49.81	
2–3	563 (53.26)	198 (35.17)	31.31–39.18	
4	412 (38.98)	106 (25.73)	21.69–30.11	
Have children				0.467
No	386 (36.52)	128 (33.16)	28.60–37.97	
Yes	671 (63.48)	208 (31.00)	27.59–34.57	
Subjective health status				0.150
Bad	53 (5.01)	21 (39.62)	27.29–53.05	
Normal	371 (35.10)	127 (34.23)	29.54–39.17	
Great	633 (59.89)	188 (29.70)	26.24–33.35	
Chronic disease				0.209
No	841 (79.56)	275 (32.70)	29.59–35.92	
Yes	216 (20.44)	61 (28.24)	22.56–34.50	
Anxiety				0.354
No	793 (75.02)	246 (31.02)	27.88–34.31	
Yes	264 (24.98)	90 (34.09)	28.57–39.96	
Depression				0.139
No	734 (69.44)	223 (30.38)	27.14–33.78	
Yes	323 (30.56)	113 (34.98)	29.94–40.30	
History of COVID-19 infection				0.146
No	85 (8.04)	33 (38.82)	28.98–49.42	
Yes	972 (91.96)	303 (31.17)	28.32–34.14	
History of COVID-19 vaccination				0.177
Not fully vaccinated	157 (14.85)	52 (33.12)	26.12–40.74	
Fully vaccinated	470 (44.47)	161 (34.26)	30.07–38.63	
At least one booster dose	430 (40.68)	123 (28.60)	24.49–33.01	
Hesitancy of COVID-19 vaccine				<0.001 *
No	849 (80.32)	197 (23.20)	20.46–26.13	
Yes	208 (19.68)	139 (66.83)	60.23–72.96	
Pandemic fatigue of COVID-19				<0.001 *
Low	455 (43.05)	92 (20.22)	16.72–24.09	
Moderate	488 (46.17)	191 (39.14)	34.88–43.52	
High	114 (10.79)	53 (46.49)	37.52–55.64	
Perceived susceptibility of influenza				<0.001 *
Low	222 (21.00)	93 (41.89)	35.54–48.45	
Moderate	500 (47.30)	155 (31.00)	27.06–35.15	
High	335 (31.69)	88 (26.27)	21.77–31.17	
Perceived severity of influenza				<0.001 *
Low	320 (30.27)	132 (41.25)	35.95–46.70	
Moderate	539 (50.99)	163 (30.24)	26.48–34.22	
High	198 (18.73)	41 (20.71)	15.51–26.75	

* A *p*-value less than 0.05 is considered to be statistically significant.

**Table 4:** Influencing factors of influenza vaccine hesitancy among reproductive-age women without past-year influenza vaccination in China.

Variables	aOR	95% CI	*p* Value
Age (ref.: <35 years)			
≥35 years	1.70	1.18–2.45	0.004 *
Location (ref.: urban)			
Rural	0.71	0.52–0.97	0.031 *
Family member (ref.: 1)			
2–3	1.78	1.03–3.10	0.040 *
4	1.44	1.05–1.97	0.023 *
Subjective health status (ref.: bad)			
Normal	0.36	0.22–0.61	<0.001 *
Great	0.25	0.16–0.40	<0.001 *
Chronic disease (ref.: no)			
Yes	0.61	0.41–0.91	0.014 *
History of COVID-19 vaccination (ref.: not fully vaccinated)			
Fully vaccinated	1.59	1.04–2.42	0.033 *
At least one booster dose	1.14	0.74–1.76	0.549
Hesitancy of COVID-19 vaccine (ref.: no)			
Yes	5.94	4.11–8.58	<0.001 *
Pandemic fatigue of COVID-19 (ref.: low)			
Moderate	2.14	1.55–2.96	<0.001 *
High	2.23	1.36–3.66	0.002 *
Perceived susceptibility of influenza (ref.: low)			
Moderate	0.57	0.39–0.83	0.004 *
High	0.46	0.30–0.72	<0.001 *
Perceived severity of influenza (ref.: low)			
Moderate	0.67	0.48–0.95	0.023 *
High	0.44	0.27–0.73	0.001 *

* A *p*-value less than 0.05 is considered to be statistically significant.

## Data Availability

All data in the study are available from the corresponding author by request.

## References

[1] World Health Organization Influenza (Seasonal). https://www.who.int/news-room/fact-sheets/detail/influenza-(seasonal).

[2] Iuliano A.D., Roguski K.M., Chang H.H., Muscatello D.J., Palekar R., Tempia S., Cohen C., Gran J.M., Schanzer D., Cowling B.J. (2018). Estimates of global seasonal influenza-associated respiratory mortality: A modelling study. Lancet.

[3] Liu Y., Liu T., Yao M., Wang Q., Li R., Kou Z. (2024). Coverage of influenza vaccination and influencing factors among healthcare workers in Shandong Province, China, 2021–2022. Hum. Vaccin. Immunother..

[4] Grohskopf L.A., Ferdinands J.M., Blanton L.H., Broder K.R., Loehr J. (2024). Prevention and Control of Seasonal Influenza with Vaccines: Recommendations of the Advisory Committee on Immunization Practices-United States, 2024–2025 Influenza Season. Morb. Mortal. Wkly. Rep. Recomm. Rep..

[5] Cao G., Guo Z., Liu J., Liu M. (2023). Change from low to out-of-season epidemics of influenza in China during the COVID-19 pandemic: A time series study. J. Med. Virol..

[6] Tao L., Wang R., Liu J. (2021). Comparison of Vaccine Acceptance Between COVID-19 and Seasonal Influenza Among Women in China: A National Online Survey Based on Health Belief Model. Front. Med..

[7] Ding H., Black C.L., Ball S., Fink R.V., Williams W.W., Fiebelkorn A.P., Lu P.J., Kahn K.E., D’Angelo D.V., Devlin R. (2017). Influenza Vaccination Coverage Among Pregnant Women-United States, 2016–2017 Influenza Season. Morb. Mortal. Wkly. Rep. Recomm. Rep..

[8] MacDonald N.E., McDonald J.C. (2014). The benefits of influenza vaccine in pregnancy for the fetus and the infant younger than six months of age. J. Paediatr. Child Health.

[9] Fell D.B., Dodds L., McNeil S., MacDonald N.E. (2014). H1N1 influenza vaccination during pregnancy. Br. Med. J..

[10] Razzaghi H., Kahn K.E., Black C.L., Lindley M.C., Jatlaoui T.C., Fiebelkorn A.P., Havers F.P., D’Angelo D.V., Cheung A., Ruther N.A. (2020). Influenza and Tdap Vaccination Coverage Among Pregnant Women-United States, April 2020. Morb. Mortal. Wkly. Rep. Recomm. Rep..

[11] Razzaghi H., Kahn K.E., Calhoun K., Garacci E., Skoff T.H., Ellington S.R., Jatlaoui T.C., Black C.L. (2023). Influenza, Tdap, and COVID-19 Vaccination Coverage and Hesitancy Among Pregnant Women-United States, April 2023. Morb. Mortal. Wkly. Rep. Recomm. Rep..

[12] Kurasawa K. (2023). Maternal vaccination-current status, challenges, and opportunities. J. Obstet. Gynaecol. Res..

[13] Offeddu V., Tam C.C., Yong T.T., Tan L.K., Thoon K.C., Lee N., Tan T.C., Yeo G.S.H., Yung C.F. (2019). Coverage and determinants of influenza vaccine among pregnant women: A cross-sectional study. BMC Public Health.

[14] Sun G., Zhang L., Qiu Y., Jia Y., Wang Y., Xu H., Zhang A., Hao L., Zhu W., Ye C. (2024). Changes of influenza vaccination rate and associated influencing factors after the COVID-19 pandemic in Shanghai, China. Hum. Vaccin. Immunother..

[15] World Health Organization Ten Threats to Global Health in 2019. https://www.who.int/news-room/spotlight/ten-threats-to-global-health-in-2019/.

[16] MacDonald N.E. (2015). Vaccine hesitancy: Definition, scope and determinants. Vaccine.

[17] Larson H.J., Jarrett C., Eckersberger E., Smith D.M.D., Paterson P. (2014). Understanding vaccine hesitancy around vaccines and vaccination from a global perspective: A systematic review of published literature, 2007–2012. Vaccine.

[18] Troiano G., Nardi A. (2021). Vaccine hesitancy in the era of COVID-19. Public Health.

[19] Lazarus J.V., Wyka K., White T.M., Picchio C.A., Rabin K., Ratzan S.C., Parsons Leigh J., Hu J., El-Mohandes A. (2022). Revisiting COVID-19 vaccine hesitancy around the world using data from 23 countries in 2021. Nat. Commun..

[20] Lin C., Tu P., Beitsch L.M. (2021). Confidence and Receptivity for COVID-19 Vaccines: A Rapid Systematic Review. Vaccines.

[21] Yang J., Atkins K.E., Feng L., Baguelin M., Wu P., Yan H., Lau E.H.Y., Wu J.T., Liu Y., Cowling B.J. (2020). Cost-effectiveness of introducing national seasonal influenza vaccination for adults aged 60 years and above in mainland China: A modelling analysis. BMC Med..

[22] (2025). Questionnaire Star. Sample Service. https://www.wjx.cn/sample/service.aspx.

[23] Kertz S., Bigda-Peyton J., Bjorgvinsson T. (2013). Validity of the Generalized Anxiety Disorder-7 scale in an acute psychiatric sample. Clin. Psychol. Psychother..

[24] Maten N., Kroehl M.E., Loeb D.F., Bhat S., Ota T., Billups S.J., Schilling L.M., Heckman S., Reingardt C., Trinkley K.E. (2021). An evaluation of clinical decision support tools for Patient Health Questionnaire-9 administration. Ment. Health Clin..

[25] Löwe B., Decker O., Müller S., Brähler E., Schellberg D., Herzog W., Herzberg P.Y. (2008). Validation and standardization of the Generalized Anxiety Disorder Screener (GAD-7) in the general population. Med. Care.

[26] Manea L., Gilbody S., McMillan D. (2015). A diagnostic meta-analysis of the Patient Health Questionnaire-9 (PHQ-9) algorithm scoring method as a screen for depression. Gen. Hosp. Psychiatry.

[27] Xin L., Wang L., Cao X., Tian Y., Yang Y., Wang K., Kang Z., Zhao M., Feng C., Wang X. (2022). Prevalence and influencing factors of pandemic fatigue among Chinese public in Xi’an city during COVID-19 new normal: A cross-sectional study. Front. Public Health.

[28] Janz N.K., Becker M.H. (1984). The Health Belief Model: A decade later. Health Educ. Q..

[29] Ghosh P.K., Chaudhry A., Campbell J.E., Kim M., Smith K., Demir F., Zhao J. (2024). Impacts of the US CDC recommendation on human papillomavirus vaccine uptake, 2010-2015. Front. Public Health.

[30] Dubé E., Laberge C., Guay M., Bramadat P., Roy R., Bettinger J.A. (2013). Vaccine hesitancy. Hum. Vaccines Immunother..

[31] Qin C., Deng J., Du M., Liu Q., Wang Y., Yan W., Liu M., Liu J. (2023). Pandemic Fatigue and Vaccine Hesitancy among People Who Have Recovered from COVID-19 Infection in the Post-Pandemic Era: Cross-Sectional Study in China. Vaccines.

[32] Wong L.P., Alias H., Siaw Y.L., Muslimin M., Lai L.L., Lin Y., Hu Z. (2022). Intention to receive a COVID-19 vaccine booster dose and associated factors in Malaysia. Hum. Vaccin. Immunother..

[33] Ali-Saleh O., Bord S., Basis F. (2022). Factors Associated with Decisions of Arab Minority Parents in Israel to Vaccinate Their Children against COVID-19. Vaccines.

[34] Du M., Tao L., Liu J. (2022). Association between risk perception and influenza vaccine hesitancy for children among reproductive women in China during the COVID-19 pandemic: A national online survey. BMC Public Health.

[35] Rosenstock I.M., Strecher V.J., Becker M.H. (1988). Social learning theory and the Health Belief Model. Health Educ. Q..

[36] Wang R., Tao L., Han N., Liu J., Yuan C., Deng L., Han C., Sun F., Chi L., Liu M. (2021). Acceptance of seasonal influenza vaccination and associated factors among pregnant women in the context of COVID-19 pandemic in China: A multi-center cross-sectional study based on health belief model. BMC Pregnancy Childbirth.

[37] Sallam M., Dababseh D., Eid H., Al-Mahzoum K., Al-Haidar A., Taim D., Yaseen A., Ababneh N.A., Bakri F.G., Mahafzah A. (2021). High Rates of COVID-19 Vaccine Hesitancy and Its Association with Conspiracy Beliefs: A Study in Jordan and Kuwait among Other Arab Countries. Vaccines.

[38] Murphy J., Vallières F., Bentall R.P., Shevlin M., McBride O., Hartman T.K., McKay R., Bennett K., Mason L., Gibson-Miller J. (2021). Psychological characteristics associated with COVID-19 vaccine hesitancy and resistance in Ireland and the United Kingdom. Nat. Commun..

[39] Domnich A., Cambiaggi M., Vasco A., Maraniello L., Ansaldi F., Baldo V., Bonanni P., Calabrò G.E., Costantino C., de Waure C. (2020). Attitudes and Beliefs on Influenza Vaccination during the COVID-19 Pandemic: Results from a Representative Italian Survey. Vaccines.

[40] Rasmussen S.A., Jamieson D.J., Bresee J.S. (2008). Pandemic influenza and pregnant women. Emerg. Infect. Dis..

[41] Cosma C., Radi A., Cattano R., Zanobini P., Bonaccorsi G., Lorini C., Del Riccio M. (2025). Exploring Chatbot contributions to enhancing vaccine literacy and uptake: A scoping review of the literature. Vaccine.

[42] Del Riccio M., Guida A., Boudewijns B., Heemskerk S., van Summeren J., Schneeberger C., Stelma F., van der Velden K., Timen A., Caini S. (2025). A Missed Opportunity? Exploring Changes in Influenza Vaccination Coverage During the COVID-19 Pandemic: Data From 12 Countries Worldwide. Influenza Other Respir. Viruses.

[43] Wang Q., Yue N., Zheng M., Wang D., Duan C., Yu X., Zhang X., Bao C., Jin H. (2018). Influenza vaccination coverage of population and the factors influencing influenza vaccination in mainland China: A meta-analysis. Vaccine.

